# Musical Interaction Reveals Music as Embodied Language

**DOI:** 10.3389/fnins.2021.667838

**Published:** 2021-07-14

**Authors:** Alessandro Dell’Anna, Marc Leman, Annamaria Berti

**Affiliations:** ^1^Department of Art, Music, and Theatre Sciences, IPEM, Ghent University, Ghent, Belgium; ^2^SAMBA Research Group, Department of Psychology, University of Turin, Turin, Italy

**Keywords:** embodied music cognition, musical joint action, predictive coding, music as language, sense of joint agency

## Abstract

Life and social sciences often focus on the social nature of music (and language alike). In biology, for example, the three main evolutionary hypotheses about music (i.e., sexual selection, parent-infant bond, and group cohesion) stress its intrinsically social character (Honing et al., 2015). Neurobiology thereby has investigated the neuronal and hormonal underpinnings of musicality for more than two decades (Chanda and Levitin, 2013; Salimpoor et al., 2015; Mehr et al., 2019). In line with these approaches, the present paper aims to suggest that the proper way to capture the social interactive nature of music (and, before it, musicality), is to conceive of it as an embodied language, rooted in culturally adapted brain structures (Clarke et al., 2015; D’Ausilio et al., 2015). This proposal heeds Ian Cross’ call for an investigation of music as an “interactive communicative process” rather than “a manifestation of patterns in sound” (Cross, 2014), with an emphasis on its embodied and predictive (coding) aspects (Clark, 2016; Leman, 2016; Koelsch et al., 2019). In the present paper our goal is: (i) to propose a framework of music as embodied language based on a review of the major concepts that define joint musical action, with a particular emphasis on embodied music cognition and predictive processing, along with some relevant neural underpinnings; (ii) to summarize three experiments conducted in our laboratories (and recently published), which provide evidence for, and can be interpreted according to, the new conceptual framework. In doing so, we draw on both cognitive musicology and neuroscience to outline a comprehensive framework of musical interaction, exploring several aspects of making music in dyads, from a very basic proto-musical action, like tapping, to more sophisticated contexts, like playing a jazz standard and singing a hocket melody. Our framework combines embodied and predictive features, revolving around the concept of joint agency (Pacherie, 2012; Keller et al., 2016; Bolt and Loehr, 2017). If social interaction is the “default mode” by which human brains communicate with their environment (Hari et al., 2015), music and musicality conceived of as an embodied language may arguably provide a route toward its navigation.

“… la domanda se la musica sia o non-sia un linguaggioè una domanda mal posta alla quale non ha senso dareuna risposta affermativa o negativa; mentre potremmotrovare *interessante* considerare la musica *alla luce* della molteplicità di aspetti presenti nell’analogia in essa suggerita.” (Giovanni Piana, *Filosofia della Musica* 1991)

Following Darwin, living beings interact with their ecosystem to create a context that ensures their own replication or (in neo-darwinian terms) the replication of their genotype (as opposed to their fenotype). On the other hand, human beings interact in such complex manners that their context, more than other animals’ contexts, transcend their genetic imperatives in favor of their contextual imperatives (Dennett, 2017). Music, like language, may be positioned exactly between these two imperatives, insofar as it is constituted by a biological component shared by every homo sapiens around the world and by a cultural component, differentiating human groups on Earth. The two components are usually called musicality and music: “Musicality in all its complexity can be defined as a natural, spontaneously developing set of traits based on and constrained by our cognitive and biological system. Music in all its variety can be defined as a social and cultural construct based on that very musicality” (Honing et al., 2015, p. 2, see also Huron, 2001). Life and social sciences often focus on the social nature of music (and language alike). In biology, for example, the three main evolutionary hypotheses about music, i.e., sexual selection (Miller, 2000; Fitch, 2006), parent-infant bond (Dissanayake, 2008; Malloch and Trevarthen, 2009) and group cohesion (Freeman, 2000; Dunbar, 2012), stress its intrinsically social character. Neurobiology thereby stresses the neuronal and hormonal underpinning of musicality. In line with these approaches, the present paper aims to suggest that the proper way to capture the social interactive nature of music (and, before it, musicality), is to conceive of it as an embodied language, rooted in culturally adapted brain structures. Here is a working definition of music as embodied language: **a means of coordination based on the sense of joint agency induced by the motor actions evoked by sounds**. This proposal heeds Ian Cross’ call for an investigation of music as an “interactive communicative process” rather than “a manifestation of patterns in sound” (Cross, 2014), with an emphasis on its embodied and predictive (coding) aspects (Vuust and Witek, 2014; Lesaffre et al., 2017). A similar attempt has been recently made by van der Schyff and Schiavio when they write that their “biocultural approach sees (musical) cognition as an emergent property of situated embodied activity within a developing socio-material environment” (2017, p. 7). We align with such a proposal, which basically holds that corporeal, neural and environmental levels concurred in shaping musical behaviors since early societies. However, in order to avoid misunderstandings, we stress the metaphorical way of using the word ‘‘language’’ in our proposal^1^. **We do not want to say that music processing works like language processing, even though the roots of both processes might be the same (**Mithen, 2005). In particular, music lacks the clear referentiality that allows us to refer to the world by means of language (if anything, music has a “floating intentionality,” as Cross put it, 2014). Nevertheless, as language, music is endowed with a syntactic, a semantic and a pragmatic aspect. We focus on the latter of this aspect and maintain that the metaphor of music as embodied language might not only better identify music as an exquisitely social phenomenon, but also encourage further investigation into the common ground it shares with linguistic competence itself.

To sum up, in the present paper our goal is: (i) to propose a framework of music as embodied language based on a review of the major concepts that define joint musical action, and with a particular emphasis on embodied music cognition and predictive processing, along with some relevant neural underpinnings; (ii) to review three experiments that provide evidence for, and can be interpreted according to, the new conceptual framework proposed in (i). In the first part we pave the way for our framework, explaining some concepts originating from both cognitive musicology and neuroscience. Our framework draws on the joint action literature and may be seen as a development of embodied and predictive coding approaches to music within such a framework. Since we are particularly interested in the neural bases of making music together, we summarize some of the more recent relevant studies, though without further elaborating on it. Our main point is to introduce a framework of music as embodied language, which is biologically plausible and keeps together the manifold evidence collected so far in the field of ensemble musicking (Small, 1998). Some of this evidence comes directly from our laboratories and it is therefore presented in the second part of the paper. Focusing on time, space and quality of musical interactions in dyads of musicians and naïve subjects, the three of our experiments aim at corroborating the view of music as embodied language based on the core concept of joint agency.

## First Part. A Framework of Music as Embodied Language

### Joint Action

Joint action has been extensively investigated in cognitive science for more than a decade. A working definition put forward by Sebanz et al. (2006, p. 70) states that a joint action is “any form of social interaction whereby two or more individuals coordinate their actions in space and time to bring about a change in the environment.” While lifting an object together has been a rather widely studied instance of joint action (Marsh et al., 2009), the change in the environment mentioned in the above definition may be at the same time subtler and deeper, as when, for instance, two persons exchange gazes in order to read each other’s intentions (Becchio et al., 2018). Actually, the need for comprehension of social interaction has been recently reiterated by a number of neuroscientists, stressing that the “social mode” is arguably the default mode of homo sapiens’ brain, not to mention other social species and mammals in general (Caccioppo et al., 2010; Schilbach et al., 2013; Hari et al., 2015). Therefore, it is urged that brain studies develop appropriate methodologies to deal not only with action observation (like classic mirror neurons paradigms), but also with contexts in which two or more subjects modulate each other’s behavior on the fly, be it for competition or cooperation. It is well known that the mirror neurons system is a brain network that is recruited similarly, not only during movement production but also during action observation suggesting its involvement in action understanding (Rizzolatti and Sinigaglia, 2010) and imitation (Iacoboni et al., 1999). An early suggestive finding in order to overcome the limits of a “spectatorial” paradigm (Reddy and Uithol, 2015) came from Newman-Norlund et al. (2007), who showed higher BOLD activation in fronto-parietal areas (which are supposed to match the human mirror neurons system) during complementary, rather than simulative, action planning (of a power or a precision grip of an object). These authors found that the very same neural network responsible for passive understanding of observed actions is active (indeed, it is more active) in (preparing) a possible interaction. Mother-infant exchanges epitomize the essence of social interaction. Indeed, this condition highlights that observation is always embedded in the dynamic processes of adaptation, reaction, incitement etc., well before any conscious awareness of the context from the infant side, portraying what De Jaegher and Di Paolo (2007) call “participatory sense-making” (see below). Hyper-scanning, the simultaneous acquisition of cerebral data from two or more subjects, provides an interesting possibility to explore social interaction, since it takes into account more than one individual at the same time, although the results imply interpretations that are far from straightforward (Konvalinka and Roepstorff, 2012; Babiloni and Astolfi, 2014; Hari et al., 2015).

### Embodied Cognition: Focusing on Its “Extended” Component

A central idea for the present work is embodied cognition, a multi-faceted theoretical paradigm that has been questioning for three decades the basic tenets of traditional cognitive science, in particular, the computational-representational nature of the mind (Varela et al., 1991; Clark, 1997; Thompson and Varela, 2001; Noë, 2004; Chemero, 2009; Gallagher, 2017). Embodied cognition stresses the entanglement of body, environmental and social components or the so-called 4E, that is, the embodied, embedded, extended and enactive components of mind and cognition (Newen et al., 2018). It is beyond the scope of the present paper to elaborate on each of these aspects^2^, but some of them need explanation in view of a theoretical effort to combine two apparently opposed music research frameworks, that is, embodied music interaction (Leman, 2007, 2016) and predictive coding (Vuust and Witek, 2014; Koelsch et al., 2019), making them converge on the concept of embodied language.

Introducing the body into the picture entails that mind and cognition are no more conceived of as building representations of the external world by means of neuronal computations, but rather as guiding processes of actions in/on the world, including parts of the world that are particularly meaningful for humans (and animals in general): conspecifics. The previous sentence highlights two of the four E we have to consider in more details, the embodied and, in particular, the extended nature of the mind. As to the former feature, we may focus our attention on what Hurley (1998) calls “the sandwich view,” according to which the core of the mind lies between perception and action, that is, in those computational processes occurring after the sensory stimuli, but before the motor responses. Embodied approaches have challenged this view, pointing out that what an organism perceives is a function of how it moves and, vice versa, how an organism moves is a function of what it perceives (as Merlau-Ponty and Gibson already put it). Such sensorimotor loops depend not only on brain processes, but also on body morphologies and functioning, insofar as different bodies may be attuned to different environmental affordances (see below). It is the interplay between brain, body and world (Clark, 1997) that allows mind and cognition to emerge, overcoming the computational and brain-centered approach of classical cognitive (neuro) science.

The “extended” component of the embodied framework is typically one of the most controversial among the 4E (Menary, 2010), since, in its strong version, it implies the inclusion of (parts of) the external world in the computational machinery an organism makes use of to solve a given cognitive problem (Clark and Chalmers, 1998’ s “parity principle”: the brain has no cognitive priority on the environment). Tool use is the classic example. When a blind cane user touches the edge of a building in order to orient himself and turn in the right direction, they say the cane becomes part of his body, as if his own fingers were sampling the environment. Now, consider a joint action like cycling together in a tandem bike. Not only is that action impossible for only one person, but the degree of synchronization necessary to accomplish it is so tight that a kind of “super-ordinate” system may emerge from the coordinated individual actions, i.e., an extended system made up by two (or more) interactive agents (Müller et al., 2018). These are two ways of extending the mind, by means of tool use, in the former case, and by means of coordination with a conspecific, in the latter. Both are relevant for music. Whatever the philosophical arguments to include such extensions in the computational machinery of the mind, the previous phenomena (tool use in particular) have been thoroughly investigated in recent cognitive neuroscience and will be briefly presented in what follows.

1) Which cognitive processes is tool use supposed to extend? Although philosophers have pointed also at memory and thought, neuroscience has focused mainly on body and peri-personal space perception. Rizzolatti et al. (1981) discovered in the ventral premotor cortex (vPC), putamen and intra-parietal sulcus (IPS) of macaque monkeys visuo-tactile bimodal neurons discharging both when an object appears close to the body and when it touches the body. Insofar as such neurons are body-part centered, codifying for the space of and around the hand, the head or the torso, they may be considered as the neural correlates of the body space (the proprioceptive and tactile space) and the peri-personal space (the multisensory space reachable by the arms). As to the body space, Graziano (1999); Graziano and Gandhi (2000) demonstrated that those neurons’ receptive fields are activated by objects in the vicinity of a fake hand (while the monkey’s real hand is occluded from view) and by the position of the fake hand, after it is embodied by means of a synchronous stimulation of both the fake and the real (occluded) hand. This is a well-known phenomenon called “the rubber hand illusion” (Botvinick and Cohen, 1998), in which a fake hand is judged as one’s own hand, if it is placed in a position congruent with one’s own body and gets synchronously touched along with one’s real (occluded) hand by means of a brush. Therefore, body ownership, the feeling that a body part is owned by a given subject, turns out to be modulated by the position, shape, and movement of the fake hand. Similarly, peri-personal space has been shown to be a plastic phenomenon. Iriki et al. (1996, see also Maravita and Iriki, 2004), indeed, demonstrated that, after practicing to collect objects with a rake, the visuo-tactile bimodal neurons of the macaque IPS extended their receptive fields to cover the entire length of the rake. In other words, while before practicing with tools such neurons discharged only when a stimulus appeared close to the hand or the shoulder or touched them, after practicing they discharged also for stimuli appearing in the far space, as far as the rake length. Such a remapping of a near space that becomes far has its equivalent in humans. For example, patients suffering from visual neglect after a stroke showed a dissociation of the near and the far space, with the neglect appearing only in the former, as assessed by means of a line bisection task. However, if the line bisection was carried out with a stick, rather than with a light-pen, thus extending the arm length, the neglect transferred also to the far space (Berti and Frassinetti, 2000; Neppi-Mòdona et al., 2007).
2) More recently, neuroscience has addressed the possibility that also social interaction has some influence on cognitive processes like body or peri-personal space perception. Soliman et al. (2015) put forward that, during and after a joint action like sawing a candle together with a string, a pair develops a “joint body-schema” that is measurable by means of a visuo-tactile multisensory integration (MSI) task. The task consists of a reaction time response to a tactile stimulus delivered either on the thumb or on the index finger while a visual stimulus appears either close to the thumb/index finger of the participant or close to the thumb/index finger of the partner (see Maravita et al., 2003 for the details). Contrary to the solo condition, during the joint condition the incongruity (e.g., thumb touched/index seen) impacted on the reaction times, slowing them down, thereby indicating that an interdependence of the two subjects’ body-schema has emerged, due to the joint action just accomplished. Taking advantage of a different MSI paradigm, Teneggi et al. (2013) show that a cooperative, compared to an uncooperative, interaction in an economic game may modulate the peri-personal space of a person in a dyad. Indeed, after the cooperative condition, subjects reacted faster to a tactile stimulus on their hands, not only when an auditory stimulus was heard close to them, but also when the sound came from a further distance, close to the cooperative partner (see Canzoneri et al., 2012 for the details). Since a response to a tactile stimulus is facilitated by an auditory stimulus in the peri-personal space, thanks to the above-mentioned bimodal neurons, this result is taken as evidence that the peri-personal space got extended, after the cooperative interaction.

At this point it is also worth stressing that Thompson and Varela (2001), two of the main theorists of embodied cognition, already argued that one of the three dimensions of embodiment is inter-subjective interaction (along with what they name “bodily self-regulation” and “sensorimotor coupling”). As we will see, one of our experiments tackles one of the previous two constitutive “extended” features of the embodied framework, exploring the multisensory peripersonal space of musicians after a (jazz) cooperative/uncooperative musical interaction (see below). However, in order to set the stage for our theoretical proposal and for each of our single experiments, we need to consider the predictive coding approach and how the embodied issues just discussed translate into musical terms.

### Predictive Coding: Focusing on Its “Sensorimotor” Component

The sensorimotor loops we described above as a crucial feature of the embodied approach strikingly resemble the “circular sensorimotor causality,” which Friston (2013) points at in presenting the predictive coding approach (see also Clark, 2016). The circular sensorimotor causality in this inferential process implies that “external states cause changes in internal states, via sensory states, while the internal states couple back to the external states through active states—such that internal and external states cause each other in a reciprocal fashion. This circular causality may be a fundamental and ubiquitous causal architecture for self-organization” (Friston, 2013, pp. 2–3). According to the predictive framework, the brain is in charge of making sense of the external world by minimizing the error resulting from the comparison between a prediction of the causes of a sensory state and such a state. Suppose that such a state is to perceive someone grasping a scalpel (Kilner et al., 2007). The brain might use its knowledge of the context (say, a hospital) as a prior to be compared with the observed action, hypothesizing that the scalpel has been grasped to cure a given patient. A big error would be forwarded to the brain prediction level, if the scalpel would be used to hit the patient’s head. On the contrary, a smaller error would be forwarded, if the scalpel would be put in a sterilization box, and no error at all would be forwarded, if the scalpel would be really used to operate the patient. In any case, the prediction error would allow updating the priors (that, once updated, become posteriors) in a continuous, circular process of sensorimotor-based predictions. The world is thus modeled in Bayesian terms as a “hierarchy of systems where supraordinate causes induce and moderate changes in subordinate causes,” offering “contextual guidance toward the most likely cause of the sensory input” (ibidem: p. 163).

Such sensorimotor loops can be further characterized as active inferences, in that the whole body, rather than the brain alone, actively enables the inferential (predictive) process, actively sampling the (external or internal) environment, conspecifics’ behaviors included. Thus, if the inferential mechanisms are read in sensorimotor, rather than computational-representational terms, the Bayesian approach can coexist and enrich the embodied approach (see Maes, 2016 and Gallagher and Allen, 2016 for sketches of similar synthesis proposal and below § 1.6). A model of music according to predictive coding principles has been recently put forward by Koelsch et al. (2019), who state that, while listening to music (even without playing it), we might generate sensorimotor predictions about rhythmical features coupled with motor actions like tapping, bobbing the head or dancing, in particular when the music “grooves.” Such predictions are continuously updated, comparing them with the actual auditory scene. In other words, moving to the music helps disambiguate some of its features by means of an embodied prediction that may be depicted in Bayesian terms. Indeed, this model, which the authors explicitly label as “enactive” (ibidem: p. 74), musical appreciation is driven not simply by error minimization, but rather by the fluctuations in the uncertainty of predictions. After reviewing some relevant literature about embodied music cognition, we will come back to these concepts in order to integrate them in a single, encompassing framework revolving around the idea of music as embodied language.

### Embodied Music Cognition

A disembodied view typically conceives of music cognition as a computational reconstruction of the hierarchical organization of music in a recursive way, from the basic acoustic stimuli to the wide formal structure of a given composition, much like a generative grammar view on language cognition (Lerdahl and Jackendoff, 1983). Embodied music cognition, on the contrary, takes advantage of the above-mentioned sensorimotor loops as a crucial feature of brain functioning to highlight the role of the body in music perception and production (Leman, 2007). This is substantiated by studies in synchronization and entrainment, in disambiguation and in outsourcing of timing (Maes, 2016).

Firstly, consider entrainment, the phenomenon that brings a body rhythm to synchronize to a music rhythm (Clayton, 2012; Phillips-Silver and Keller, 2012; Moens and Leman, 2015). The sensorimotor prediction and adaptation mechanisms are supported by neuronal circuits in the posterior parietal lobe, premotor cortex, cerebro-cerebellum and basal ganglia, giving rise to the phenomenon of “groove” (Janata et al., 2012), suggesting that the same processes that cause bodily motion are involved in musical rhythm perception. As Todd writes: “If the spatiotemporal form of certain [sensory] stimuli are matched to the dynamics of the motor system, then they may evoke a motion of an internal representation, or motor image, of the corresponding synergetic elements of the musculoskeletal system, *even if the musculoskeletal system itself does not move*” (Todd, 1999, p. 120). Iyer (2002) emphasizes that music may evoke different human actions according to its tempo, like breathing, walking and speaking (with frequencies, respectively, between 0,1 and 1 HZ, between 1 and 3 HZ, between 3 and 10 HZ), but the other way around is also true. Indeed, much existing music compositions lie in this tempo range, suggesting that bodily resonators have somehow modeled the way humans create music (van Noorden and Moelants, 1999).

Secondly, movement can also disambiguate a metric structure. In a couple of experiments Phillips-Silver and Trainor (2005) let infants be passively bounced or adults bend their knees to an ambiguous rhythmic pattern. These subjects’ oscillations were set to stress either the second or the third beat, thus rendering either a binary or a ternary meter, as was manifest by their answers afterward, when asked to recognize which of two different patterns they moved on (while the adults answered verbally, the infants were observed attending to their preferred pattern between those two).

Thirdly, timing is often not a matter of counting but rather a matter of moving, using outsourcing strategies by which limbs are moved, or choreographies are maintained in loops that don’t require cognitive attention. Su and Pöppel (2012) showed that non-musicians rely more than musicians on their own movement in order to feel the pulse of a rhythmic sequence, missing it when such movements are not allowed. However, musicians can also rely on their internal clock to understand the sequence even without moving, thus demonstrating the importance of body movement, in particular where expertise is absent. In addition, it is worthwhile to remind that mirror neurons have been shown to depend also on such a sensorimotor expertise. For example, inferior-frontal and parietal areas typically involved in mirror activation, have been found to be more active (in a fMRI scan) in pianists, compared to naïve subjects, while observing piano-playing, compared to non-piano-playing, finger movements (Haslinger et al., 2005, see also Herholz and Zatorre, 2012).

A framework proposed by Leman (2007) holds that: “The human body can be seen as a biologically designed mediator that transfers physical energy up to a level of action-oriented meanings, to a mental level in which experiences, values, and intentions form the basic components of music signification. The reverse process is also possible: that the human body transfers an idea, or mental representation, into a material or energetic form” (ibidem, p. xiii). The physical energy is the acoustic surface of music and the corresponding mental representation is the intention attributed by the listener/producer to that music, “on the basis of a simulation of the perceived action in the subject’s own action” (ibidem, p. 92, see also Koelsch et al., 2019 as summarized above). In other words, through a repertoire of motor actions (both transitive and intransitive, i.e., gestures), the body maps musical features like rhythm, melodic contours, intensities, tempi etc., promoting their understanding and enjoyment. While Schiavio and Menin (2013) interpret this approach as dualism, Leman’s proposal can best be understood differently, with mind (involving a certain form of attention and a reflection upon signification) as an emerging by-product of body-related processes (Broeckx, 1981). Indeed, the mediation between the mental and the physical occurs at a conscious level of processing, typically involved with reflective meaning-formation, whereas much processing can be conceived of in a strictly sensorimotor way, suspending any commitment about the nature of what the body is assumed to mediate. Consider again the disambiguation process allowed by moving a body part according to either a binary or a ternary meter on an isochronous pulse. There is no need to attribute physical properties to the sound beats we hear and, on the other hand, mental properties to our subjective experience to the extent that the perception of those sounds is coupled to the body movements necessary to disambiguate them. What counts for an embodied approach to (music) cognition is that exactly such sensorimotor loops, rather than abstract computations, constitutes (music) cognition. Importantly, the sensorimotor mechanism we are dealing with here is twofold. On the one hand, it concerns body morphology, the fact that the human body allows for different actions from other animal bodies, for example, as we saw above, synchronizing around specific frequency ranges, according to the motor action involved. On the other hand, sensorimotor mechanisms have a specific neural counterpart, well represented by the mirror network in both humans and monkeys (Rizzolatti and Sinigaglia, 2008 and below).

Mirror neurons further clarify how multi-person music interactions can be understood as embodied processing that cannot be reduced to internal processes of mindreading or simulation of interacting brains (Thompson and Varela, 2001). Following De Jaegher and Di Paolo (2007), we could rather talk of “participatory sense-making” (see Schiavio and De Jaegher, 2017, for a musical application of this concept), pointing to the embodied feature of an interaction provided by the continuous negotiation of spatiotemporal parameters between two (or more) subjects. De Jaegher and Di Paolo (2007) draw our attention on a very basic joint action like passing together through a door that is too narrow to let two subjects enter at the same time without bending and adjusting to the size and position of each other’s body. Note that, if these persons were asked to repeat that action many times, they would likely do it every time in a slightly different manner, thus making it evident that a slightly different dynamics of mutual adjustment unfolded, though resulting in the same outcome (passing through the door). If we apply this scenario to an ensemble music context, some features may emerge that are tightly related to the temporal connection of bodies and sound relationships in spaces.

In Walton et al. (2015), for example, the forearm and head movements of two pianists improvising either on a drone track (a uniform alternation of two chords) or on an ostinato track (a complex four chords progression) were recorded by a motion capture system. Thanks to cross wavelet transform (CWT), the time series of these movements revealed different periodicities, according to the features of the musical track. The ostinato track, indeed, repeated every 4 s, allowing the musicians’ movements to coordinate, as it turned out, at multiples of 4 seconds. On the contrary, the drone track didn’t show any specific periodicity, probably due to its simpler structure, that offered the musicians more variety of motion (and, as a consequence, of musical possibilities, but the opposite is also true). The authors drew the conclusion that expressive interactions are guided not only by brain processes, but also by bodily dynamics emerging on the fly, in accordance with one of the tenets of the embodied approach to cognition (see also Walton et al., 2018).

### Neural Sensorimotor Underpinnings of Musical Interaction

In the last decade, a number of studies have investigated the neural circuits that enable musical joint action. Evidence for the involvement of M1 in action observation was provided by Fadiga et al. (1995)’s pioneering research, in which a subject cortico-spinal activation was shown to enhance while looking at a transitive motor action (grasping) of another person over an object, compared to simply looking at that object (see Aziz-Zadeh et al., 2004 for an auditory counterpart). At least two studies have corroborated such finding in the ensemble music domain. In the first study, Novembre et al. (2012) let a sample of pianists rehearse a couple of compositions before asking them to play their melody part with their right hand either alone or while the left hand part was being played by a (hidden recorded) partner. Single-pulse TMS on the inactive left hand/arm M1 showed higher motor-evoked potentials (MEPs) in the ensemble condition, highlighting that motor representations may arise in response to potential social interaction. In a second study, Novembre et al. (2014) tested another sample of pianists, half of which had rehearsed and the other half of which had not rehearsed a given tune. When asked to adapt the tempo of their right hand to the gradually changing tempo of the left hand played by a partner, after double-pulse TMS on left hand M1, the latter group showed higher accuracy than the former. That is, double-pulse TMS disturbed only those processes relying on the sensorimotor simulation of the rehearsed part played by the partner, a mechanism that is clearly recruited in the real-time coordination of actions generated by the self and the partner.

Given the fact that musicality refers to the biological component, it can be expected that we find sensorimotor mechanisms like the ones just described also in non-musicians. For example, Gordon et al. (2018) recently found cortico-spinal facilitation in non-musicians FDI while they were looking at a three-note piano sequence with sound lagging 200 ms behind the video, compared to a correct audio-visual stimulus condition or to the correct unimodal conditions (either visual or auditory). The authors conclude that sensorimotor predictive models are here at stake, rather than simulation-like mechanisms, given that only the violation of the expected sensory outcome caused an increase in cortico-spinal excitability. On the other hand, if non-musicians are trained to execute simple melodies at the piano, when listening to those melodies their FDI cortico-spinal excitability increases even some milliseconds before the tone onset, thus showing the difference a motor training makes, compared to simply listening (Stephan et al., 2018). This finding is consistent with Candidi et al. (2012), who demonstrated that pianists manually trained with a given composition exhibited higher fingers MEPs than pianists only visually trained with that composition, whenever an incorrect piano fingering was observed.

Again, these findings can be interpreted as sensorimotor processes that support an embodied approach to music processing in combination with a Bayesian predictive processing approach (see next paragraph). Multimodal sensorimotor neurons are likely the substrate of such processes, in particular in areas like STG and STS, which respond more strongly to auditory-visual stimuli than to auditory or visual stimuli separately (Beauchamp et al., 2004, see Kohler et al., 2002 for echo neurons). However, as we hinted at above, interactive brain research methods are paving the way to overcome some of the constraints that characterized social neuroscience since the mirror neurons discovery. A pioneering study has been Babiloni et al. (2012), which explored the musical performance of three different saxophone quartets by means of simultaneous EEG, discovering that alpha rhythms in frontal areas (Bas 44/45) are correlated with empathy scores in musicians who are observing their own performance (about musical hyper-scanning see also Osaka et al., 2015 and Pan et al., 2018).

### A Framework for Music as Embodied Language

**FIGURE 1:**
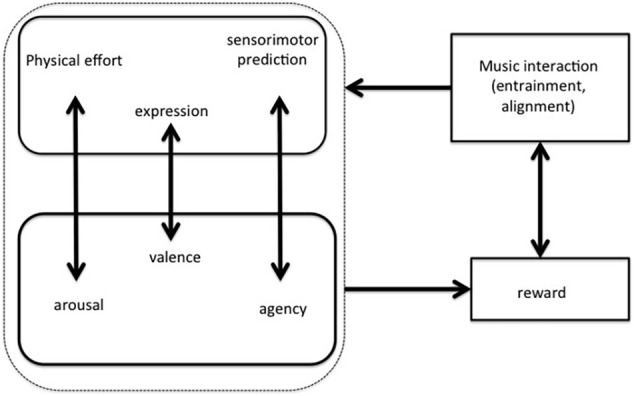
The interaction-reward hypothesis (Leman, 2016) states that a rewarding interaction with music (through entrainment and alignment) is based on physical effort, cognitive control, and expressive gesturing that match with arousal, agency, and pro-social values.

The above theoretical strands, based on embodiment and predictive processing, should be complemented by an emotional-motivational layer that involves reward outcomes (Salimpoor et al., 2015). As such, every kind of interaction with music (be it listening or playing, be it alone or in group) can be understood as constituted by a cognitive-motivational loop that realizes reward and empowerment in the subjects involved in it. Therefore Leman’s (2016) recent model includes physical effort, predictive processing and expression (as biosocial signals) in parallel with arousal, agency and pro-social attitudes (Figure 1).

We will see in a moment whether and how this model could be integrated with Keller et al. (2014, Keller, 2008)’ s, but now we need to elaborate on only one of its components, that is, the pro-social orientation induced by the sense of agency (induced, in its turn, by the sensorimotor predictions inherent in) interacting with music, since it is crucial for our definition of music as embodied language. The sense of agency, a widely studied phenomenon in the cognitive neurosciences, is the feeling of control of a given person on a given action he/she is accomplishing (Haggard and Eitam, 2015). In everyday life it is an implicit feeling, which becomes manifest if something goes wrong, as when you are on the point of pressing a light switch, but the light turns on the instant before you press it: it is not you, who turned on the light, but someone else, hence a weak (or totally absent) sense of agency. On the other hand, being probably built on the prediction of our action consequences, rather than on their real sensory consequences (Berti and Pia, 2006), an illusory sense of agency may also ensue.

Sensorimotor predictions (based on the above-mentioned Bayesian inferences) are able to induce the feeling that a given musical pattern has been produced by a motor action of ours, which is reminiscent of Hume’s concept of causality (Leman, 2016). Such a feeling would be (consciously) illusory in cases of moving to the music without playing it, as in running, dancing, or even simply tapping to the music, but it would be veridical whenever we are really playing the music. Nevertheless, in both cases a rewarding and empowering effect might ensue, due also to a pro-social element (valence) that (at least partly) explains the expressive power of musical interactions. This idea is consistent with accounts that emphasize the capacity music exhibits of making persons being (Overy and Molnar-Szakacs, 2009) or keeping (McNeill, 1995; Hove and Risen, 2009) together in time, developing a joint sense of agency, a concept on which the philosopher Pacherie (2012) has recently investigated (see below). Arguably, what is still missing from such a theory (as from many other proposals in the neuroscience and musicology literature) is a more detailed characterization of the relationship between expressive quality and pro-social aspects in musical interaction. Insights toward such a link can be found in Overy and Molnar-Szakacs (2009)’ Shared Affective Motion Experience (SAME) model, which “suggests that musical sound is perceived not only in terms of the auditory signal, but also in terms of the intentional, hierarchically organized sequences of expressive motor acts behind the signal” (ibidem, p. 492). Not surprisingly, these authors invoke the recruitment of the mirror neurons network as the neural implementation of such experiences with music. Furthermore, they employ the concept of “sense of agency” (differently from the standard use) to stress the sense of human interaction lying at the core of musical experience, “a sense of the presence of another person, their actions and their affective states” (ibidem, p. 494, see also Clarke, 2005; Livingstone and Thompson, 2009; Windsor and de Bézenac, 2012).

Precisely the idea that a person is lurking behind a musical sound leads to the possibility to conceive of music as an embodied language. This idea resonates with Leman’s proposal, when he claims that “musical expression is more than just a habit or settled practice. Expression locks into the biology of human social interaction behavior, where it is easily linked up with affective states and attitudes” (2016: 49). Unlike natural language, music allows to coordinate in real time behaviors of big size groups, as epitomized in stadium choirs or in war and work songs, and it is well known, particularly in ethnological studies, how such behaviors enhance collective identities, that is, cultural membership (Freeman, 2000; Nettl, 2005; Clarke et al., 2015). If biological traits of musicality (underlying music) are likely met in tonal encoding of pitch, beat perception and metrical encoding of rhythm (Honing et al., 2015), we may think they underlie the communicative character of musicality and, as a consequence, of music as an embodied interactive communicative process (Mithen, 2005; Malloch and Trevarthen, 2009; Cross, 2014). Therefore, we may expect to find these traits uniformly distributed among humans, no matter how musically expert they are, representing the prerequisites for musical expertise, rather than its outcome (Mehr et al., 2019).

In order to show how an embodied language might work, let’s again consider ensemble music, a sophisticated form of joint action that, perhaps not surprisingly, has allowed for about a decade a balanced study between controlled experimental conditions, on the one hand, and ecologically valid setting, on the other hand (D’Ausilio et al., 2015). According to Keller’s model (Keller, 2008; Phillips-Silver and Keller, 2012; Keller et al., 2014), interpersonal coordination in a music ensemble relies on a combination of higher-order cognitive processes, like sharing a global idea of the musical composition at stake (which, in turn, depends on socio-cultural conventions), and lower-order cognitive-motor competences, like mutual adaptive timing, prioritized integrative attending and anticipatory imagery. These processes may somehow characterize every kind of joint action (Vesper et al., 2010), but in a musical context they amount to the fact that:

1) two or more subjects need their temporal playing coordination be so tight and flexible to cope with unintentional micro-perturbation of timing, due to the intrinsic variability of human actions, and, on the other hand, with intentional timing variations due to expressive purposes (accelerando/ritardando). Phase and period correction are two mechanisms put forward to explain such competences (Repp and Su, 2013).
2) A musician needs to pay attention not only to what he is playing, but also to what the ensemble is playing, prioritizing his resources for the former process, without losing track of the latter. Internal time-keepers have been postulated to keep track of the multi-layered structure of ensemble music, being it quite often composed of rhythmic sections, intertwined melodic lines and, more generally, different parts according to the performance/composition (London, 2004).
3) Musicians need to anticipate their partners’ playing to some extent, if they aim at keeping their performance stable and coherent. Keller and Appel (2010) demonstrated, for example, that the most synchronized among a few piano duets were those formed by pianists with higher imagery vividness in a task of notes continuation without auditory feedback.

The predictive coding approach seems able to unify all the previous three aspects, since music is endowed with an intrinsically hierarchical structure from both the melodic (cells within phrases within sections) and rhythmic (beats within cells within meters) viewpoint, that Bayesian inference can suitably tackle (Salimpoor et al., 2015; Koelsch et al., 2019). Moreover, also musical interaction can profit from such a framework. Indeed, the sensorimotor loops necessary for an individual action to take place, predicting the outcome of a given action and adjusting it in case of wrong sensory feedback, can be translated into social terms (Wolpert et al., 2003; Kilner et al., 2007; Friston and Frith, 2015; Volpe et al., 2016; Brattico and Vuust, 2017). In the latter case, we may predict the consequences of an action of ours on a partner (say, accepting to be kissed), while the sensory feedback would be provided by the partner’s reaction (say, avoiding us), which, in turn, allows for an adjustment of our action (say, pretending to reach something just behind the partner) to minimize prediction errors. In a musical context, let this action be the attack of the theme after seven introductory measures of the jazz standard *Autumn Leaves*. The musician who plays the theme must adapt to the tempo set by the rhythm section (say, piano, bass, and drums), attending to its own sound without neglecting the others’ and predicting their correct unfolding. After playing the first two notes in the eight measure the soloist realizes that neither the bass nor the piano changed the chord leading to the real first measure of the tune, therefore he adjusts his trajectory, turning those two notes in a sort of ornament preceding the theme, whose beginning is postponed for a measure. It is worthwhile to stress that such processes need not be fully aware, since internal models are supposed to work in a nested hierarchy, from very low levels (close to reflexes) to conscious levels (very close to propositional thought, see Friston and Frith, 2015). As we will see, two of our experiments explore adaptive timing in search of ensemble proto-musical competences in non-musicians and how they are modulated by the embodiment of a partner’s hand, or looking for dynamic markers of a singing performance quality that matches subjective reports about joint agency and about that very quality (see below).

**FIGURE 2:**
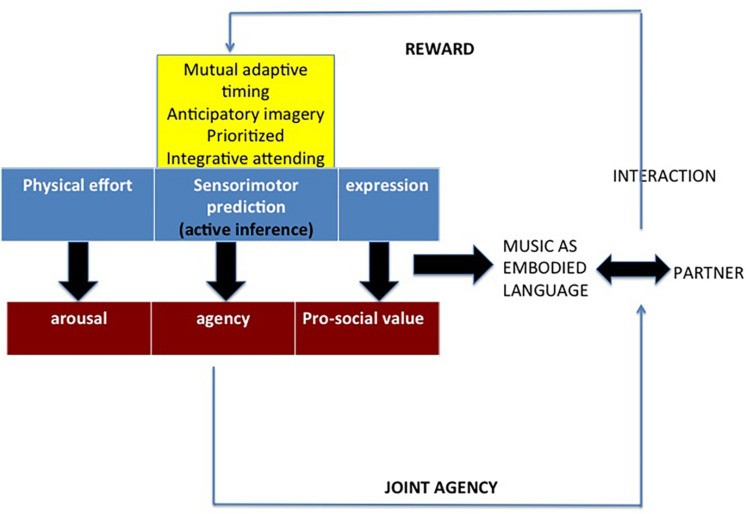
A framework of music as embodied language. The crucial role is played by joint agency, which is a consequence of the sensorimotor prediction device, along with arousal (both arousal and joint agency are expected to cause rewarding effects). The prediction devise is based on active inference and includes Keller’s three sensorimotor competences to play music in ensemble. Some experimental evidence (concerning, for example, tapping, jazz, hocket, and tool use) can be interpreted according to this framework (see below).

The concept of music and musicality as an embodied language around which the present work revolves can now be summarized by means of a diagram (Figure 2). The ambition of such a framework would be to integrate predictive coding and embodied approaches (e.g., Leman, 2007, 2016; Keller, 2008; Vuust and Witek, 2014; Keller et al., 2014; Koelsch et al., 2019). The new framework involves three components necessary to play music together. A first component, including Keller’s three sensorimotor competences, deploys active inference during a musical interaction, be it an individual or a joint action, be it simply listening or producing music. A second component involves agency as a consequence of embodied sensorimotor predictions. A third component involves arousal as an ensuing feature.

Joint agency is how we see agency in a musical context. Indeed, given the pro-social value of musicality (and, then, of music) as an embodied language (Wiltermuth and Heath, 2009; Kokal et al., 2011), agency in such contexts is not simply the feeling of being in control of a given individual action, as in grasping an object for an ordinary action, but it has to be characterized by a “joint” component, implying the more or less evident presence of one or more musically interacting subjects. The weak version of this framework is easily applied to a real ensemble performance as the dyadic interactions we are going to explore in the following experiments, but a stronger version would identify a social component also in individual interactions with music. Indeed, if music (and, before it, musicality) is the bio-cultural product of social interactions, it might be argued that a track of its social origin is always present, regardless of the kind of musical interaction at stake. The most obvious example is ensemble music, but we may draw a scale of decreasingly evident presence of another agent in listening to music (less, if it is live music, more, if it is not) and, eventually, in playing alone. From this point of view, playing alone may be compared to speaking alone, either when rehearsing a monolog or when thinking aloud. The resulting sense of joint-agency would decrease accordingly (see also Clarke, 2005; Livingstone and Thompson, 2009; Overy and Molnar-Szakacs, 2009; Windsor and de Bézenac, 2012 for similar accounts). As for the third component of the network, we simply consider the following aspects. An experiment such as Fritz et al. (2013) has shown that whenever a person controlled some parameters of music by means of various gym tools her feeling of exertion was lower compared to a passive condition, in which she simply listened to music while doing gym. Moreover, the motivating force of music has been shown in several experiments investigating walking speed to music compared to metronome (Styns et al., 2007) or to different musical genres (Leman et al., 2013), identifying genres that appear more activating than other genres. In all these cases a transfer of sonic energy to motor energy seems to be happened (see also Tarr et al., 2014).

## Second Part. Experimental Evidence

Three of our own experiments described in the next sections provide evidence for, and can be interpreted in the given framework. They have a focus on the time, the space and the quality of the musical interaction, respectively. In a first experiment, we show that also non-musicians may proto-musically communicate. We investigated the timing of their joint tapping and whether and how it is modulated by the position of the partner (with the relative cortico-spinal activation measured by means of single-pulse TMS). In a second experiment, we show that the peripersonal space of two interacting jazz musicians may be modulated according to the cooperative or uncooperative character of such interaction, measuring such a space by means of a MSI paradigm that allowed us also a comparison between musicians and non-musicians’ reaction times. Finally, in the last experiment, we explicitly focus on the concept of joint agency in hocket dyads, correlating such subjective parameter with an objective and dynamic measure of their timing quality, devised according to Bayesian principles.

### Time: Entrainment and Embodiment in a Tapping Interaction

**FIGURE 3:**
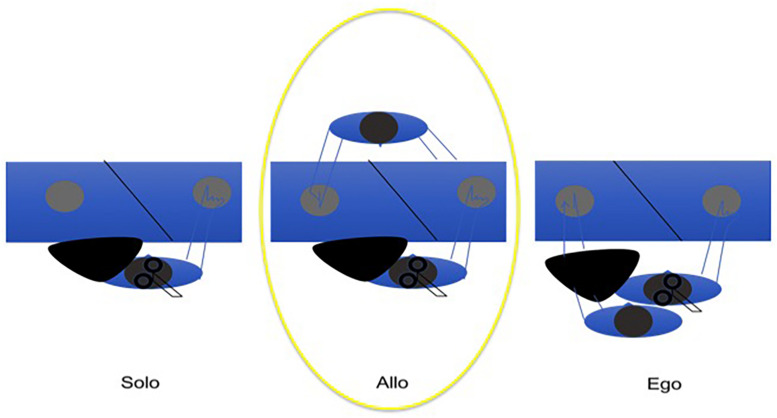
The allocentric condition induces higher cortico-spinal activation than (but comparable joint-correlation of asynchronies to) the egocentric condition, resulting in the better condition for joint agency.

An easy way to investigate mutual adaptive timing is tapping, a proto-musical motor action allowing also non-musicians to align a body part movement to the beat of the music. Previous experiments have shown that musicians are able to adapt their timing to the timing of the partner’s tapping in anti-phase (Nowicki et al., 2013), and that non-musicians are able to do the same in an in-phase tapping task (Konvalinka et al., 2010). Under the assumption of an innate musicality, similarly to Konvalinka et al. (2010, see also Koelsch et al., 2000), we have shown (Dell’Anna et al., 2018) that also non-musicians are able to entrain to the timing of their partner in an alternate (i.e., anti-phase) joint tapping task to a reference metronome providing half-cycle ticks (i.e., at zero and half phase of the cycle). Given a regular reference, we used the correlation of asynchronies as a method to measure entrainment. The alternate tapping task has been carried out in three conditions: (i) alone with the metronome, (ii) with a partner in front of the subject and (iii) with a partner beside the subject, in a position congruent with his/her body such that the partner tapped with the left hand, while the subject tapped with the right hand (Figure 3). The latter condition exploits the paradigms where ‘alien’ hands can be incorporated both in healthy subjects (where a rubber hand is felt as one’s own, given particular manipulations and constrains, the rubber hand illusion, Botvinick and Cohen, 1998, see also above) and in brain damaged patients (where a real hand belonging to someone else is felt and believed to be the own hand, Garbarini et al., 2014). We expected to see higher cortico-spinal excitability in condition (ii), compared to condition (i) and (iii), due to mirror mechanisms that the shared action should activate. When the distinction between the self and the other becomes weaker, as in condition (iii), then the mirror mechanism doesn’t act, as if there is no longer any partner to interact with, and therefore, cortico-spinal excitability will be similar between (i) and (iii). The results of our experiment show that timing is mutually adaptive in condition (ii) and (iii), but not in (i). In addition, there is a difference in ownership between (ii) and (iii) because in the latter condition the alien hand is felt as the own hand, with a feeling of agency over the tapping. In condition (iii), when the subject embodies an alien hand, cortico-spinal excitability tends to decrease, compared to condition (ii) when there is a partner in front (Schutz-Bosbach et al., 2006; Della Gatta et al., 2016). The results can be interpreted as if an interaction context sets the motor system to be engaged, while an embodied partner (‘s hand or arm) results in no social interaction. MEPs recording by means of TMS on M1 first dorsal interossus (FDI) confirmed this idea in our proto-musical task. When the tapping subject embodied the partner’s arm (as assessed by subjective reports of agency and ownership), cortical excitability did not differ from the alone condition. On the contrary, when the partner tapped in front of the partner, the sociality of the context brought about higher cortico-spinal excitability, in accordance also with the mirror neurons literature (Fadiga et al., 1995; Novembre et al., 2012). Working as part of an embodied language, the rhythmic component provided by the metronome mutually entrained the basic motor actions of the interacting dyad, before any conscious awareness of the process from the subjects’ side.

### Space: Remapping of Peripersonal Space in a Jazz Interaction

**FIGURE 4:**
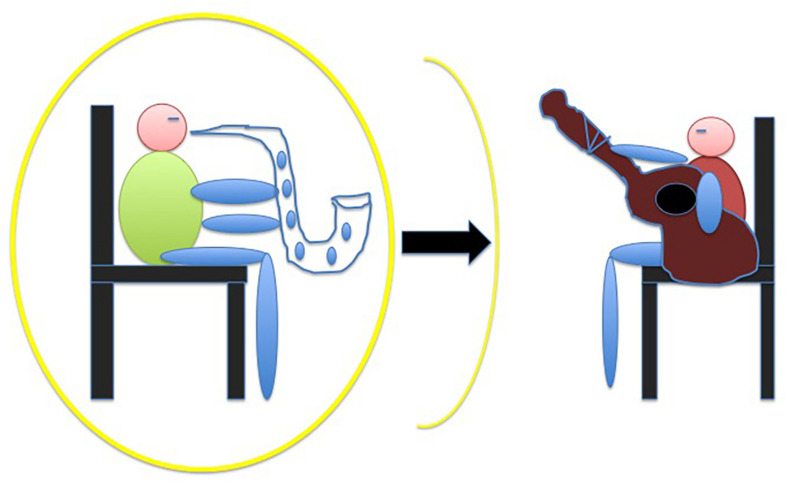
We expected an extension of the musician’s peripersonal space after the cooperative interaction, but we obtained a sort of disappearance of it after the uncooperative interaction. In any case, we may conclude that the joint sense of agency was corrupted by the uncooperative partner.

As mentioned, the peripersonal space, which is the multisensory body-part-centered representation of the space immediately surrounding the body, has been recently shown to be sensitive not only to tool use (Iriki et al., 1996; Berti and Frassinetti, 2000), but also to social interaction (Patané et al., 2016; Pellencin et al., 2018). In particular, peripersonal space has been shown to extend after a cooperative economic exchange compared to an uncooperative economic exchange (Teneggi et al., 2013). Likewise, we let pairs of musicians play with a partner playing either the correct or the incorrect harmonic sequence of a jazz standard tune, under the hypothesis that only the former condition would have caused an extension of the musicians’ peripersonal space (Figure 4). In order to measure peripersonal space after the two experimental conditions (the cooperative and the uncooperative harmonic condition) we borrowed an audio-tactile integration task devised by Serino et al. (2007, see also Canzoneri et al., 2012) who showed that a sound occurring close to the subject, compared to a far sound, facilitates reaction times to a co-occurring tactile stimulus. A far sound is thereby influenced by what is subjectively experienced as far. It turned out (Dell’Anna et al., 2020b), by contrast, that only the uncooperative condition, influenced the size of the peripersonal space, making it disappear, as if the subject withdrew from the uncooperative partner. We interpreted this result as evidence that, insofar as music and musicality are intrinsically social embodied languages, a musical interaction has a measurable impact on the perception of the space between two (or more) subjects. The paradigm allowed us also to compare our sample of musicians with a sample of non-musicians. Coherently with a recent finding (Landry and Champoux, 2017), we confirmed that musicians are faster than non-musicians in reacting to audio-tactile stimuli, regardless of the distance of the auditory stimulus, arguably due to the musicians’ sensorimotor training with their instrument and (to a lesser extent) singing, that brings about well-known cortical-subcortical reorganizations (Munte et al., 2002; Zimmerman and Lahav, 2012).

### Quality: Expressive Timing and Joint Agency in A Hocket Interaction

**FIGURE 5:**
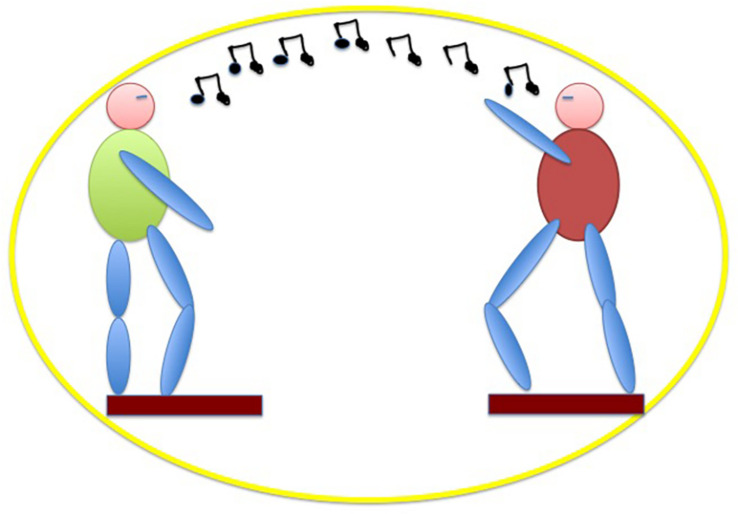
The joint agency is strengthened whenever subsequent inter-onset-intervals are correctly predicted by the Bayesian system the singing couple builds up.

The experiments described so far, although concerning musical interaction, are focused on an event-based analysis approach. A central aim of another of our experiments was to devise a way to capture the dynamics of a singing dyad in order to assess the interaction quality of a hocket performance, focusing on timing (Dell’Anna et al., 2020a). While the main part of studies on timing in pairs of musicians have used some form of correlation of asynchronies or mean signed asynchronies (Goebl and Palmer, 2009; Clayton et al., 2019), the methods remain event-based due to the fact that references that occur are regular time instances, which can be interpreted as subsequent events. Here we tried to develop a method that could cope with the intrinsic variability of human behavior over time, regardless of a fixed reference. In fact, given the alternate nature of hocket singing, the reference is latently available (as an emerging tempo that can possibly change over time). To account for interaction, we chose the inter-onset intervals between any two notes (coming from two notes sang by two singers in sequence) and computed in Bayesian terms a duration error, relative to the time-varying latent tempo that we used as predictor for the duration. This approach, where the latent tempo is a sort of moving average that is used as predictor for measuring the subsequent observed inter-onset- interval resulted in a dynamic measure of timing accuracy, which we called fluctuation error. Since we were also interested in the subjective experience of a musical interaction, we correlated such measure of timing with self-assessment of the performance quality and feeling of joint agency reported by the singers after the performance (Figure 5). Recently, there has been an intense debate about the concept of joint agency. According to Pacherie (2012), there is a SHARED and a WE sense of joint agency (Dewey et al., 2014; Bolt et al., 2016; Bolt and Loehr, 2017), the former being the feeling of controlling part of the joint action, the latter being the feeling of blending with the partner in a single entity while accomplishing that action. Indeed, a singing dyad can be conceived of as a dynamical Gestalt whose components constrain each other’s unfolding performance by means of that embodied language represented by music (Walton et al., 2015; Müller et al., 2018). The way we built our hocket score could have caused a WE-agency, but in fact a SHARED-agency was found. Moreover, we discovered higher correlation for self-annotation than joint agency values with respect to duration errors.

### Application of the Framework

Echoing Cross’s (2014) call for investigating music as a “communicative interaction process” and combining predictive code with embodied accounts of music, we now formulate an interpretation of the previous three experiments in the light of our framework of music as an embodied language. In the first experiment, musicality, rather than music, is considered, as long as only the isochronous pulse of the metronome and the entrained beats of the pair tapping on the drumpads constituted the acoustic pattern, hence the proto-musical interaction, as we named it. The embodied character of the musical language is here given by its capacity to coordinate at a micro-timing level the motor actions of the two interacting subjects, despite their lack of musical expertise. Importantly, according to our framework, both the prediction of the partner’s next (couple of) taps and the ensuing feeling of joint agency count as part of that cognitive-motivational loop instantiated by the musical interaction. On the contrary, the “joint” component of agency does not seem to play any role in either Keller’s or Leman’s above-mentioned models. The correlation we found between the partners’ s asynchronies may be interpreted as a first approximation of a timing marker of such embodied language, that is, of a feature that identifies a more or less successful expressive exchange between two (or more) subjects. On the other hand, we think we have found another and more interesting timing marker in our study on the quality of hocket singing. The interest of this marker lies in its dynamic nature, which takes advantage of the continuous reciprocal adjustment between the two singers’ s tone onsets on the bases of short-term predictions. In particular, in this study we focused on a timing feature, the inter-onset-interval between two singers’ tones, which is intrinsically inter-subjective, thus somehow already applying the concept of embodied language. Contrary to the other two experiments, the hocket study explicitly investigated also the joint agency parameter, finding a correlation between it and the dynamic marker of the performance timing quality, which further corroborates the view of music as embodied language based on joint agency. There again, the result of the study on peripersonal space modulation after a jazz interaction may be understood as the effect of a lack of joint agency. The uncooperative condition, indeed, altered the “mutual incorporation” (Fuchs and De Jaegher, 2009) necessary to coordinate a musical joint action not only in time, but also in (peripersonal) space, thus preventing reward and empowerment. This is a clear example of a failed (embodied) communication that somehow breaks the superordinate system in place whenever a musical ensemble interaction unfolds (causing the explosion of the temporary bubble surrounding the musicians, metaphorically speaking, but see Bufacchi and Iannetti, 2018 for a criticism of such a metaphor). To conclude, although the three sensorimotor competences posited by Keller (2008, Keller et al., 2014), that is, mutual adaptive timing, anticipatory imaging and prioritized integrative attention, and the components of Leman’s model Leman (2016), that is, prediction, physical effort and expressive signaling, are certainly at work in our three experimental scenarios, we emphasize the crucial role of another factor, that is, joint agency. The feeling of a shared control over a given (musical) action or the complete unity with one or more conspecifics allowed by a musical performance in the form of rites, ceremonies or simply mother-infant exchanges, constitutes such an important feature of music and musicality as embodied languages that further research is surely needed to disentangle all its complexities.

As we said, while the application of our framework to social contexts seems quite straightforward, its strong version should consider individual interactions with music as well. An example is the following study currently underway in our laboratories. Since in our first experiment we used TMS in order to confirm the sociality of the allocentric condition and in our second experiment we used audio-tactile MSI as a proxy for measuring peripersonal space, in a new study we are combining both methods. We aim at investigating whether a wind musician’s cortico-spinal activation is enhanced by touching a trumpet while listening to trumpet tones (compared to touching a scissors and/or listening to white noise), under the hypothesis that his/her expertise would induce him/her to feel prepared to act with another (virtual) musician, should the congruent audio-tactile condition take place (see Schulz et al., 2003 and Yamaguchi et al., 2014 for inspiring empirical data). To this aim we are comparing a sample of wind musicians with a sample of non-musicians, insofar as only the former group is expected to show such a form of joint agency marker, due to the specific competence required by the experimental context and, arguably, by the underlying mirror neurons circuits (see also above). In other words, the conflation of the auditory and the tactile stimulus might be a function of overlearned expertise, given that for the performer the act of engaging with the trumpet in the first place is necessarily confounded with the sound of that instrument.

## Conclusion

To sum up, in this paper we drew on both cognitive musicology and neuroscience to outline a comprehensive framework of musical interaction, taking advantage of several aspects of making music in dyads, from a very basic proto-musical action, like tapping, to more sophisticated contexts, like playing a jazz standard and singing a hocket melody. Our framework combines insights of embodied and predictive coding approaches, centring around the concept of joint agency. If social interaction is the default mode by which humans communicate with their environment (Hari et al., 2015), music and musicality conceived of as an embodied language may arguably provide a route toward its navigation. The metaphorical character of the analogy we propose between music and language should encourage, in our opinion, further exploration of the social nature of every kind of interaction with music. Moreover, it could invite in-depth analysis of aspects other than the pragmatic one we have stressed in the present paper, starting, for example, from the mirror neuron literature about linguistic processes to highlight deeper connections between music and language (Rizzolatti and Arbib, 1998; Arbib, 2013).

To begin with, given the recent interest of neuroscience in understanding social interaction, we explored some ideas in the research context of joint action, in which embodied and predictive approaches may be better framed together. We have stressed, then, the embodied and the extended components of embodied cognition, since these may be the main features at the service of a possible integration between the two above-mentioned approaches in the musical domain. Afterward, the sensorimotor component of the predictive coding paradigm has been highlighted, in that it can be considered as the most naturally close to the embodied framework. A foray into the intensely debated domain of embodied music cognition has been proposed as a necessary step toward the outline of our synthesis, just before a brief overview of the most recent cognitive neuroscience results concerning social musical interaction. A framework of music as embodied language has been sketched, eventually, which aims at doing justice to the intrinsically interactive nature of musical experience, independently of the real social interaction that could be at stake. Joint agency, the main feature of our account of music as embodied language, is put forward as the conceptual hub around which embodied and predictive approaches may converge.

The main merit of our proposal lies in the attempt to unify different strands of research that have been highly debated in the last twenty years, and apply a new synthesis of them in the domain of music cognition. Again, to conceive of music as an embodied language means to take seriously the current neurobiology in its emphasis on the importance of social interaction in the emergence of human mind and cognition (Caccioppo et al., 2010; Dennett, 2017), part of which is constituted by the magnificent phenomenon of music. If this is correct, future research pathways should take into account that the best way to frame music is social interaction, even if we are dealing with apparently neutral features like timbre, rhythm, melodic profile and so on (see also McDermott, 2009; Bryandt, 2012).

Still, a number of limitations remain in the present work. First of all, what we are presenting is a framework, rather than a model, of music cognition, hence the difficulty of making more circumscribed hypotheses. In particular, more empirical research is needed to test how far the concept of joint agency can reach, for example, if it can really play a role also in individual interactions with music, as we posit. Second, a deeper account of the integration of several other aspects of embodied and predictive approaches that we have not discussed here seems possible and desirable. Proponents of the 4E framework, for example, invite us to include embodied cognition in the wider paradigm of “enaction” as originally proposed by Varela et al. (1991) and more recently sharpened by Gallagher (2017) or Newen et al. (2018). It would be worthwhile to examine such a possibility, since an enactive attempt in the musical domain has been persistently pursued in the last years by Schiavio and Altenmüller (2015); Schiavio and De Jaegher (2017) or van der Schyff and Schiavio (2017). Lastly, though predictive coding has been sometimes presented as compatible with embodied approaches, even by some of its proponents (Friston and Frith, 2015; Koelsch et al., 2019), the main part of its applications are brain-centered, taking advantage of the neural hierarchies easily identifiable in the brain. We have just began to show how this approach may be “extended” in the environment, not only by means of (musical) tools, but also (and primarily) by means of social interactions. A great amount of research is needed to complete the picture. Given the complex nature of the phenomenon of “musicking” together (Small, 1998), encompassing biological and cultural aspects, it will not be surprising to see increasing interdisciplinary efforts in the near future, that put together evolutionary biologists, neuroscientists, psychologists, musicologists, philosophers as well as musicians. The present work aims to be but a drop in this sea, whose boundaries remain unexplored.

## Data Availability Statement

The original contributions presented in the study are included in the article/supplementary material, further inquiries can be directed to the corresponding author.

## Author Contributions

AD elaborated the theory and wrote the draft of the manuscript. ML contributed to the elaboration of the theory and revised the manuscript. AB revised the manuscript. All authors contributed to the article and approved the submitted version.

## Conflict of Interest

The authors declare that the research was conducted in the absence of any commercial or financial relationships that could be construed as a potential conflict of interest.

## Footnotes

1 We thank two reviewers for their insistence on the inadequacy of using the word “language” literally, rather than metaphorically. See also our opening quotation from Piana (1991).

2 The embedded and enactive components will not be discussed because they would require another paper, which would be worthwhile writing in the future, as we hint at in the Conclusion.
